# Rates of marine warming onset affect reef fish mortality

**DOI:** 10.1038/s42003-020-01388-0

**Published:** 2020-11-03

**Authors:** Caitlin Karniski

**Affiliations:** Communications Biology, https://www.nature.com/commsbio

## Abstract

While much of the work examining the ecological effects of marine warming events focuses on the magnitude and duration of elevated temperatures, a recent study from Amatzia Genin and colleagues investigates how the rate of onset of warming affects the mortality of reef fish in the Red Sea. These authors document fish mortality following two warming events with dramatic increases in temperature and report that piscivores and benthic grazers were disproportionately represented among the found carcasses. Many of these fish were infected with a bacterial pathogen following the warming event. This study points to the rate of warming increase as a critical parameter to be considered when assessing the ecological effects of marine warming events, including those for which the peak temperature is not anomalous.

Marine heatwaves (MHWs) have been prevalent disruptors of marine communities in recent years, affecting both lower-trophic level sessile organisms like seagrasses and corals as well as top-order predators like bottlenose dolphins. These discrete, but prolonged, events of extreme temperature have resulted in altered community structure, regime shifts, and widespread habitat loss. While much of the work examining the ecological effects of warming events focuses on the magnitude and duration of elevated temperatures, a recent paper investigates how the rate of warming onset affects reef fish mortality.

This study^1^, led by Amatzia Genin (currently at the University of Queensland), examines rates of fish mortality following a warming event in the northern Red Sea. During this warming event in July 2017, sea surface temperatures rose by 4.2 °C in 2.5 days, the steepest increase in temperature since daily recordings began 32 years ago. A second steep incline followed two weeks later. While the maximum temperatures reached were themselves unremarkable, the unprecedented rate of warming was followed by unusual levels of fish mortality, as observed from citizen science recordings. The authors found that piscivores and benthic grazers were disproportionately represented in fish carcasses found during this period. They also found that all necropsied fish were suffering from bacterial infection at their time of death, which the authors suggest may have been induced by lower immune response and increased susceptibility to pathogens resulting from the rapid temperature increase.

Genin et al. also retrospectively assess two past reports of warming events and suggest that the rate of warming onset has also likely been responsible for fish mortality elsewhere. This study emphasizes the rate of warming increase as a critical parameter to be considered for warming events, in addition to peak temperature and duration. It also highlights the effects of warming events that are not necessarily considered marine heatwaves based on their maximum temperature.


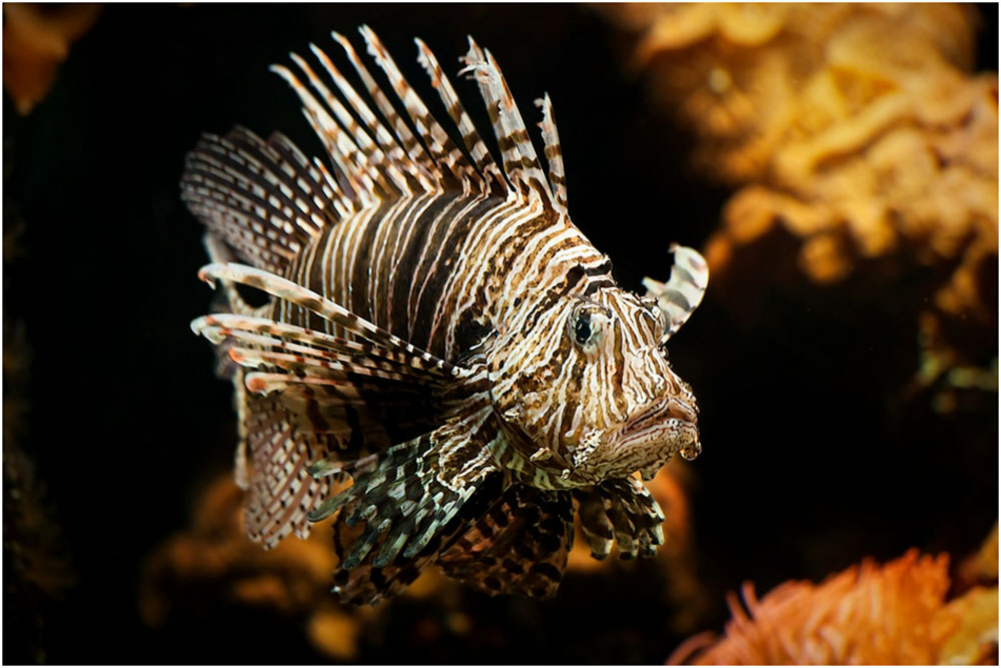


--
